# The *drnf1* Gene from the Drought-Adapted Cyanobacterium *Nostoc flagelliforme* Improved Salt Tolerance in Transgenic *Synechocystis* and *Arabidopsis* Plant

**DOI:** 10.3390/genes9090441

**Published:** 2018-09-04

**Authors:** Lijuan Cui, Yinghui Liu, Yiwen Yang, Shuifeng Ye, Hongyi Luo, Baosheng Qiu, Xiang Gao

**Affiliations:** 1 Hubei Key Laboratory of Genetic Regulation and Integrative Biology, School of Life Sciences, Central China Normal University, Wuhan 430079, China; cui7lijuan@hotmail.com (L.C.); lyh8423@hotmail.com (Y.L.); ywyang235@hotmail.com (Y.Y.); hyluowh@hotmail.com (H.L.); 2 Shanghai Agrobiological Gene Center, Shanghai 201106, China; ysf@sagc.org.cn

**Keywords:** terrestrial cyanobacteria, *Nostoc flagelliforme*, abiotic stress, transgenic study, salt-tolerant genes

## Abstract

Environmental abiotic stresses are limiting factors for less tolerant organisms, including soil plants. Abiotic stress tolerance-associated genes from prokaryotic organisms are supposed to have a bright prospect for transgenic application. The drought-adapted cyanobacterium *Nostoc flagelliforme* is arising as a valuable prokaryotic biotic resource for gene excavation. In this study, we evaluated the salt-tolerant function and application potential of a candidate gene *drnf1* from *N. flagelliforme*, which contains a P-loop NTPase (nucleoside-triphosphatase) domain, through heterologous expression in two model organisms *Synechocystis* sp. PCC 6803 and *Arabidopsis thaliana*. It was found that DRNF1 could confer significant salt tolerance in both transgenic organisms. In salt-stressed transgenic *Synechocystis*, DRNF1 could enhance the respiration rate; slow-down the accumulation of exopolysaccharides; up-regulate the expression of salt tolerance-related genes at a higher level, such as those related to glucosylglycerol synthesis, Na^+^/H^+^ antiport, and sugar metabolism; and maintain a better K^+^/Na^+^ homeostasis, as compared to the wild-type strain. These results imply that DRNF1 could facilitate salt tolerance by affecting the respiration metabolism and indirectly regulating the expression of important salt-tolerant genes. *Arabidopsis* was employed to evaluate the salt tolerance-conferring potential of DRNF1 in plants. The results show that it could enhance the seed germination and shoot growth of transgenic plants under saline conditions. In general, a novel prokaryotic salt-tolerant gene from *N. flagelliforme* was identified and characterized in this study, enriching the candidate gene pool for genetic engineering in plants.

## 1. Introduction

Environmental abiotic stresses, such as drought, salinity, and high and low temperatures, are detrimental factors limiting the growth of less tolerant organisms, including soil plants. Generating transgenic plants with abiotic stress tolerance-associated genes is a promising way to alleviate growth inhibition and crop loss [1,2]. Three major groups of genes are reported to be associated with abiotic stress responses: those involving signaling cascades and transcriptional regulation; those involving the protection of membranes and proteins; those involving water and ion uptake and transport [2,3]. Excavation of new gene resources and elucidation of their physiological or cellular roles in stress response or tolerance is still a critical issue in current plant biology. Angiosperm resurrection plants and halophytes have come to be considered excellent potential sources for exploring these genes in recent years [4,5]. However, it also deserves to be noted that only a few prokaryotic genes are commercially applied in transgenic crops, although numerous genes associated with plant stress resistance have been identified and characterized [6]. This so-called trans-boundary expression (transfer of prokaryotic genes into plants) is thus highly appreciated by some experts.

Cyanobacteria are cosmopolitan prokaryotic microorganisms that can be found from temperate to extreme habitats. In xeric regions, much of the success of cyanobacterial species is related to their ability to withstand long-term desiccation and frequent dehydration-rehydration cycles [7]. Desiccation-tolerant cells implement structural, physiological, and molecular mechanisms to survive severe water deficit [8,9]. At the same time, the periodic loss of water from cells leads to alterations of intracellular ion composition and concentration, imposing potential ion toxicity [10]. Thus, there is some overlap in the adaptation of cyanobacteria to desiccation stress and salt stress. *Nostoc flagelliforme*, a filamentous nitrogen-fixing cyanobacterium, is distributed in arid or semi-arid steppes of the west and northwestern parts of China [11]. Its habitats are characterized by high evaporation rates, substantial temperature differences, and intense solar radiation [11]. This species may serve as a valuable prokaryotic biotic resource for gene excavation [6]. However, so far only limited abiotic stress-responsive genes or proteins have been isolated or identified in it [12,13,14,15,16]. Thus, excavation of new abiotic stress-tolerant genes from *N. flagelliforme* is still needed, for expanding the candidate gene pool for transgenic use.

In our previous study, we isolated some osmotic stress-responsive complementary DNA (cDNA) fragments, and identified three of the predicted genes, named as drought resistant genes of *Nostoc flagelliforme* (*drnf*) 3, 5, and 9, respectively [14,16]. In the preliminary test via the transgenic *Escherichia coli* strain, another candidate cDNA fragment was found to be most efficient in conferring salt tolerance, named as *drnf1*; its full gene sequence (NCBI accession no. JX417708.1) was obtained according to the reported partial genome sequences [17]. The deduced DRNF1 protein harbors a conservative P-loop NTPase (nucleoside-triphosphatase) domain and a helix-turn-helix domain (CD-search, NCBI). The family of P-loop NTPases participates in various cellular processes, including translation, transcription, replication and repair, intracellular trafficking, membrane transport, and activation of various metabolites [18,19]. Several P-loop NTPases were reported to be involved in stress response and regulation in bacteria or plants, including SufC [20], Fap7 [21], SKD1 [22], and Hsp100/ClpB [23]. Recently, a rice P-loop NTPase YchF1 was reported to function as a negative regulator in abiotic stress and plant-defense responses [24]. As a potential new P-loop NTPase, the biological function (most possibly in salt tolerance) of DRNF1 awaits further investigation. In this study, we mainly investigated its salt-tolerant roles by over-expressing the *drnf1* gene in the model cyanobacterium *Synechocystis* sp. PCC 6803 and then evaluated its transgenic application potential in plants by over-expressing it in the model plant *Arabidopsis thaliana*.

## 2. Materials and Methods

### 2.1. Organisms and Culture Conditions

*Nostoc flagelliforme* was collected in 2012 in Inner Mongolia, China, and stored in an air-dried state (~11% water content) before use. The dry sample was cultured in Blue Green-11 (BG11) medium at 25 °C and continuous illumination of 40 µmol photons·m^−2^·s^−1^ overnight to recover physiological activity [16]. A Photosynthesis II (PSII) activity parameter, the ratio of variable to maximal chlorophyll fluorescence (Fv/Fm), was used to indicate the recovery or inhibition of the photosynthetic apparatus or its functional changes under stress conditions [25]. In *N. flagelliforme*, the changing trends of Fv/Fm upon abiotic stresses were positively correlated with those of the photosynthetic rates [25,26,27,28]. The Fv/Fm was detected using a Plant Efficiency Analyzer (PEA, Hansatech Instruments Ltd., King’s Lynn, UK) as described by Reference [26]. *Synechocystis* sp. PCC 6803 (hereafter referred to as *Synechocystis*) was kept in our laboratory, which was used as a cyanobacterial host for the overexpression of the *drnf1* gene. *Synechocystis* cells were statically cultured in BG11 medium in a 250 mL conical flask at 30 °C and continuous illumination of 40 µmol photons·m^−2^·s^−1^. The cultures were gently shaken two or three times per day. The model plant *Arabidopsis thaliana* ecotype Columbia (hereinafter referred to as *Arabidopsis*) was cultured in a growth chamber set at 22 °C, which was used as a plant host for overexpressing the *drnf1* gene. Plant cultivation was performed as described by Reference [29].

### 2.2. Salt Stress Treatment of Nostoc flagelliforme and drnf1 Transcription

The physiologically fully recovered *N. flagelliforme* samples were cultured in BG11 solutions supplemented with 0.2, 0.4, and 0.6 M NaCl, respectively. After 27 h, the samples were collected and washed three times with sterilized water, followed by sub-culture in normal BG11 solution for 20 h. The changes in Fv/Fm were detected during the whole cultivation process. To detect the *drnf1* transcription upon salt stress, the recovered samples were incubated in 0.4 M NaCl solution for 24 h, and at different time points samples were collected for total RNA isolation. The samples were ground in liquid nitrogen and RNAs were extracted with Trizol reagent (Invitrogen, Karlsruhe, Germany) according to the manufacturer’s instructions. DNA contaminant was eliminated by incubating the RNAs at 37 °C with RQ1 RNase-free DNase (Promega, Fitchburg, MA, USA) for 1 h. Reverse transcriptions were performed with Super-script II Moloney Murine Leukemia virus reverse transcriptase (Promega) with random hexamer primers. For semi-quantitative reverse transcription PCR (RT-PCR), 1 µL of the reverse transcription reaction was used for PCR in a final volume of 20 µL. The primers for the *drnf1* gene were *drnf1*-F (5′-ATGAATTTTAGTGCTGATCC-3′) and *drnf1*-R (5′-TTAAGATGCAATCGCTAAATC-3′). The 16S rRNA gene was amplified as an internal reference, with the primers being 16S-F (5′-CAGGTGGCAATGTAAGTCT-3′) and 16S-R (5′-TCGTCCCTCAGTGTCAGTT-3′) [26].

### 2.3. Transgene in Synechocystis and Salt Stress Treatments

Genomic DNA of *N. flagelliforme* was extracted by the cetyl trimethylammonium bromide (CTAB) method [26]. The *drnf1* sequence was amplified by PCR with the primers *drnf1*-F and *drnf1*-R. The PCR fragment was ligated into pMD18-T vector (Takara, Dalian, China) for sequencing. The resulting plasmid was inserted with an *omega*-P*_rbcL_* fragment containing spectinomycin-resistant gene and *rbcL* promoter in the *Sal*I site (ahead of the *drnf1* gene). The spectinomycin resistance cassette was excised from the plasmid pRL57 [30]; the *rbcL* promoter was amplified by PCR from *Synechocystis* as described by Reference [31]. Furthermore, the *omega*-P*_rbcL_*-*drnf1* fragment was sub-cloned into the plasmid pKW1188, which harbors a neutral integrative platform [32]. The resulting pKW1188::*omega*-P*_rbcL_*-*drnf1* was transformed into *Synechocystis* as described by Reference [32]. The spectinomycin-resistant transformants were selected and confirmed by PCR using the gene-specific primers. The transcription of *drnf1* in transgenic *Synechocystis* was confirmed by semi-quantitative RT-PCR as mentioned above.

For salt stress experiments, both short- and long-term treatments were performed. In the former, *Synechocystis* cells of exponential phase were transformed into 20 mL glass tubes contained with NaCl solutions of 0.8, 0.9, and 1.0 M, respectively. The Fv/Fm values were detected during a 3 h incubation and their relative changes were calculated. For the latter, *Synechocystis* cells were statically cultured in BG11 solutions in 250 mL conical flasks supplemented with 0, 0.9, and 1.0 M NaCl, respectively. The cultivation was performed for 10 days and the flasks were gently shaken two or three times per day. Their growth curves were evaluated by detecting the chlorophyll *a* (Chl *a*) concentration at different days. For Chl *a* extraction, 4 mL of cell suspensions was collected and mixed with 4 mL of 95% ethanol overnight. The concentration was determined as described by Reference [33].

### 2.4. Assay for Photosynthetic and Respiration Rates

Photosynthetic O_2_ evolution was measured using a Clark-type oxygen electrode (Hansatech DW1, King’s Lynn, UK) as described by Reference [34]. The respiratory activity was determined by measuring O_2_ consumption in the dark. *Synechocystis* cells at late exponential period were centrifuged at 4000× *g* for 5 min and sub-cultured in fresh BG11 solution overnight. Furthermore, 2 mL of cell suspension was centrifuged and the pellet was re-suspended in the same volume of BG11 solution. The suspensions were immediately subjected to the measurement of photosynthetic and respiration rates. For salt stress treatment, 50 mL of cell suspension was added with 0.5 M NaCl (final concentration) and incubated for 2.5 h, followed by measuring photosynthetic and respiration rates.

### 2.5. Exopolysaccharide Determination

The exopolysaccharide (EPS) is usually organized in two distinct types: capsular EPS (CPS), which is strongly bound to the external cell surface; released EPS (RPS), which is released into the surrounding environment [35]. The CPS and RPS concentrations (relative to Chl *a* concentration) were determined for *Synechocystis* cells before and after salt stress. The total EPS is the sum of CPS and RPS. The Chl *a* concentration was determined as mentioned above. The polysaccharide concentration was determined using the phenol-sulfuricacid method [36]. *Synechocystis* cells were statically cultured into the exponential period with a Chl *a* concentration of 3–4 µg/mL and subjected to polysaccharide determination. Ten milliliters of cell suspension was centrifuged at 6500× *g* for 5 min and the supernatant was directly used for RPS determination. The pellet was extracted with 10 mL of hot water (95 °C) for 2 h and after centrifugation, the CPS that already dissolved in the supernatant was determined. For salt treatments, the above-mentioned cell cultures were supplemented with 0.5 M or 0.8 M NaCl (final concentration) and continued to cultivate for five days. Then the Chl *a*, CPS and RPS concentrations were similarly determined. The EPS/Chl *a* ratios were calculated for comparative analysis. Three replicates were performed.

### 2.6. Transcriptional Analysis

*Synechocystis* cells were cultured in BG11 solution supplemented with 0.8 M NaCl and cell suspensions of 40 mL at the exponential period were collected for RNA extraction and subsequent quantitative PCR (qPCR). Reverse transcription was performed as mentioned above. qPCR was performed using SYBR^®^ Green Real-Time PCR Master Mixes according to the manufacture’s protocol (Toyabo, Osaka, Japan). The genes for qPCR analysis included glucosylglycerol (GG) synthesis-related *ggps* and *ggpp* genes [37], Na^+^/H^+^ antiport-related *nhaS1* and *nhaS3* genes [38], a response regulator gene *rre37* [39], and an RNA polymerase sigma factor gene *sigB* [40]. The primers are as follows: *ggpp* (*slr0746*-F, 5′-GATTGGGGAAATCACGGTAA-3′; *slr0746*-R, 5′-AACGCCGCTACATATTGGTC-3′), *ggps* (*sll1566-*F, 5′-ATTACGTGAAGGGCACCAAG-3′; *sll1566*-R, 5′-TTAATTTTCCCTGCCAGTCG-3′), *nhaS1* (*slr1727*-F, 5′-ATTGCCTTTCCCCTTTCCTA-3′; *slr1727*-R, 5′-AAATAGGCTCTCCCCTTCCA-3′), *nhaS3* (*sll0689*-F, 5′-TTGCCTCTGGCAGACTTTTT-3′; *sll0689*-R, 5′-AACCGGTAACCACCTTACCC-3′), *rre37* (*sll1330*-F: 5′-GCCGTGATTGATTCTGACCT-3′; *sll1330*-R, 5′-AAAATTCCTGCATGCCAAAG-3′), *sigB* (*sll0306*-F, 5′-ATGGTAACAGTGACAGTTAT-3′; *sll0306*-R, 5′-GCTTCAATCATTTTCCGTTT-3′). Transcriptional levels of target genes were normalized to those of the internal reference gene *rnpB* according to a standard process of ΔCt method [41].

### 2.7. Na^+^ and K^+^ Content Determination

Na^+^ and K^+^ contents of *Synechocystis* cells were measured by atomic absorption spectrometry. In brief, *Synechocystis* cells of the exponential period that cultured in BG11 solution supplemented with 0.8 M NaCl were collected and washed five times with deionized water. After centrifugation, the pellets were dried at 80 °C for 72 h in an oven and then weighed. The dried samples were digested in 2 mL of 70% (*v/v*) nitric acid overnight and subjected to Na^+^ and K^+^ determination using a Perkin Elmer AAnalyst 800 atomic absorption spectrometer (Shelton, CT, USA) [29].

### 2.8. Transgene in Arabidopsis, Salt Stress Treatments, and Phenotypic Observation

For construction of the expression vector, PCR-amplified *drnf1* fragment was ligated into pMD18-T vector and sub-cloned into the plasmid pBIm, under the control of the constitutive cauliflower mosaic virus 35S promoter [29]. The resulting pBIm-35S::*drnf1* was transferred to *Agrobacterium tumefaciens* strain GV3101 and then transformed into *Arabidopsis* plants using a floral-dip method [42]. Seeds were surface-sterilized by soaking in 70% ethanol for 1 min and 5% (*w*/*v*) NaClO for 5 min, followed by five washes with sterilized water. Furthermore, seeds were sown on 1/2 Murashige and Skoog (MS) medium supplemented with 50 μg/mL of kanamycin for germination. T1 generation plants that survived on the medium were confirmed by genomic PCR and semi-quantitative RT-PCR. Homozygous T3 generation lines were obtained by subsequent self-crossing and three lines with different *drnf1* expression levels were chosen for salt stress treatment and phenotypic observation. 

Salt-tolerant phenotypes of transgenic *Arabidopsis* were evaluated on seed germination and plant growth aspects. For the former, surface-sterilized seeds were sown on the plates containing 1/2 MS with 2% sucrose media in the presence of different concentrations (0, 100, 150, and 175 mM) of NaCl. After vernalization at 4 °C for four days, seed germination was conducted for 14 days in the growth chamber and germination profiles were observed with regard to the cotyledon opening [29]. Germination rates were scored. For the latter, seeds after vernalization were sown in pots filled with the vermiculite, and three-week old plantlets were subjected to first salt stress treatment. The pots were watered with 1/2 MS solution containing 150 mM NaCl and 10 days later watered again. Plant growth, especially the shoot elongation, was compared between wild-type and transgenic plants during the salt stress period of around 20 days.

## 3. Results

### 3.1. Transcriptional Induction of drnf1 by Salt Stress in Nostoc flagelliforme

**Figure 1:**
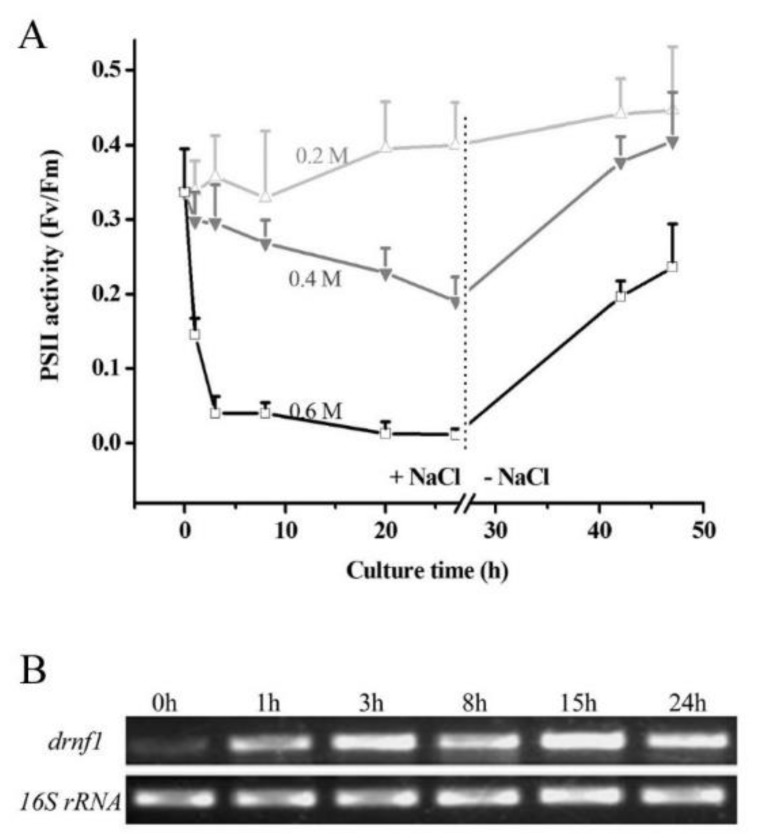
Salt stress response of *Nostoc flagelliforme* and induction of *drnf1* transcription. (**A**) Physiologically recovered samples were treated by NaCl solutions for 27 h and then transferred to normal solutions for physiological recovery for 20 h. Physiological changes were indexed with the Fv/Fm parameter. Data are mean ± s.d. (*n* = 5); (**B**) Semi-quantitative RT-PCR analysis of transcription levels of *drnf1* gene under 0.4 M NaCl for different times. *16S rRNA* gene was used an internal reference.

It was reported that the photosynthetic activity of *N. flagelliforme* increased with increased salt concentration, exhibited a maximum at 0.15 M NaCl, and dropped to zero in 0.9 M NaCl solution [11]. The physiological response of the *N. flagelliforme* sample to salt stress was further evaluated in terms of PSII activity parameter Fv/Fm (Figure 1A). NaCl at a level of 0.2 M seemed not to inhibit the sample, while 0.4 M and 0.6 M NaCl remarkably impaired the PSII activities of the samples, with the latter being more severe. After the removal of salt stress (0.4 or 0.6 M NaCl), the impaired samples could achieve complete or partial PSII activity recovery during the subsequent culture. These results were suggestive of the good recovery capability of *N. flagelliforme* from salt stress. Furthermore, transcriptional induction of *drnf1* at 0.4 M NaCl was assayed by semi-quantitative RT-PCR (Figure 1B). *drnf1* transcription remarkably increased at 1 h and reached a higher level at 3 h; during the subsequent hours, *drnf1* transcription was still maintained at an appreciable level, as compared to the control (0 h). Therefore, *drnf1* is a consistently salt-responsive gene in *N. flagelliforme*.

### 3.2. Increased Salt Tolerance in Transgenic drnf1 Synechocystis

**Figure 2:**
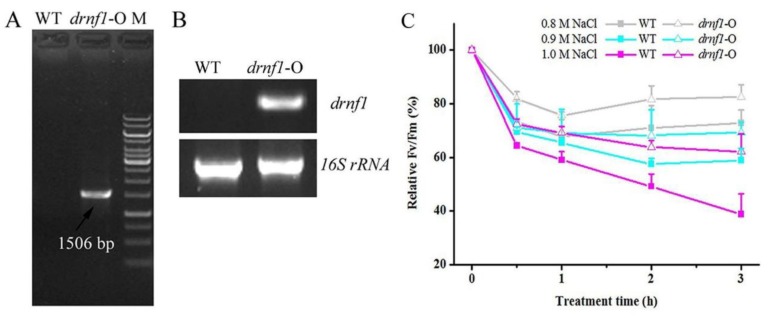
Identification of transgenic *drnf1 Synechocystis* and its resistance to short-term salt stress. (**A**) Identification of the transgenic strain using *drnf1* gene-specific primers by PCR. WT: wild-type strain. *drnf1*-O: transgenic strain. M: DNA marker; (**B**) Semi-quantitative RT-PCR detection of *drnf1* transcription in the transgenic strain; (**C**) Changes of relative Fv/Fm during salt stresses for 3 h. Data are mean ± s.d. (*n* = 5).

**Figure 3:**
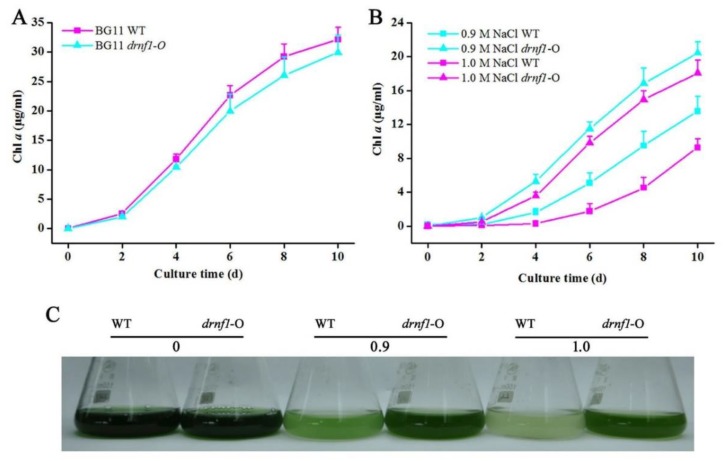
The growth performance of transgenic *Synechocystis* upon long-term salt stress. Strains were cultured under 0, 0.9 and 1.0 M NaCl conditions for 10 days. (**A**) The growth curves (Chl *a* content) of WT and transgenic *Synechocystis* strains in media without salt stress; (**B**) The growth curves (Chl *a* content) of both strains under salt stress conditions. Data are mean ± s.d. (*n* = 3); (**C**) The appearances of WT and transgenic cultures in triangular flasks at 10 days.

*N. flagelliforme* is difficult to be genetically manipulated at present. Thus, heterologous expression of the *drnf1* gene was manipulated in the model cyanobacterium *Synechocystis* as described in the Methods section. *drnf1* has no similar sequences in the genome of *Synechocystis*. As shown in Figure 2A,B, the *drnf1* sequence was integrated into the genome of *Synechocystis* and was effectively transcribed. Salt tolerance of transgenic *Synechocystis* was further evaluated by short- and long-term experiments, as compared to the wild-type (WT) strain (Figure 2C and Figure 3). In the former, three treatments (0.8, 0.9, and 1.0 M NaCl) all led to a rapid reduction in relative Fv/Fm of both strains during the initial 1 h, but this reduction was slower in the transgenic stain; along with the extension of the treatment time, both strains were inclined to achieve a stable lower Fv/Fm, whereas the transgenic strain showed significantly higher Fv/Fm levels than the WT, being 13~60% higher at 2 and 3 h (Figure 2C). The slower reduction or better maintenance in relative Fv/Fm suggested the greater stability of the PSII reaction center or better physiological status of transgenic *Synechocystis* in coping with salt stress. In the latter, the transgenic strain showed a similar growth rate (in terms of Chl *a* concentration) to the WT in normal growth conditions (Figure 3A), whereas under both 0.9 and 1.0 M NaCl conditions the transgenic strain showed a clearly faster growth (Figure 3B). Like our previous study [43], the changes of Chl *a* concentration were positively correlated to those of the optical density at 750 nm (data not shown). The appearances of cell cultures at 10 days also indicted these changes (Figure 3C). *Synechocystis* is a moderately salt-tolerant species [44]. Therefore, a further improvement of salt tolerance in it indicated that *drnf1* is a powerful gene associated with salt resistance. 

### 3.3. Photosynthesis and Respiration of Transgenic Synechocystis upon Salt Stress

Photosynthesis and respiration provide energy and metabolites for the growth. The rates of photosynthesis or respiration were compared between WT and transgenic *Synechocystis* in presence or absence of salt stress (Table 1). Under either normal or stress conditions, the transgenic strain seemed to possess slightly higher net photosynthetic rates than the WT, while respiratory rates were significantly higher in the former. It was also noted that gross photosynthetic rates were significantly higher in the transgenic strain in both conditions. The inhibition of salt stress on photosynthesis and respiration of either WT or transgenic strains was obvious, consistent with the previous report [45]. The reduction extents of photosynthesis for WT and transgenic strains were 33.4% and 33.6%, respectively, whereas the reduction extents of respiration for them were 27.2% and 22.1%, respectively. These results suggest that a basal level of photosynthesis and respiration was affected by the transgene, but the transgenic stain could sustain more active respiration metabolism under salt stress.

### 3.4. Exopolysaccharide Production in Transgenic Synechocystis

**Figure 4:**
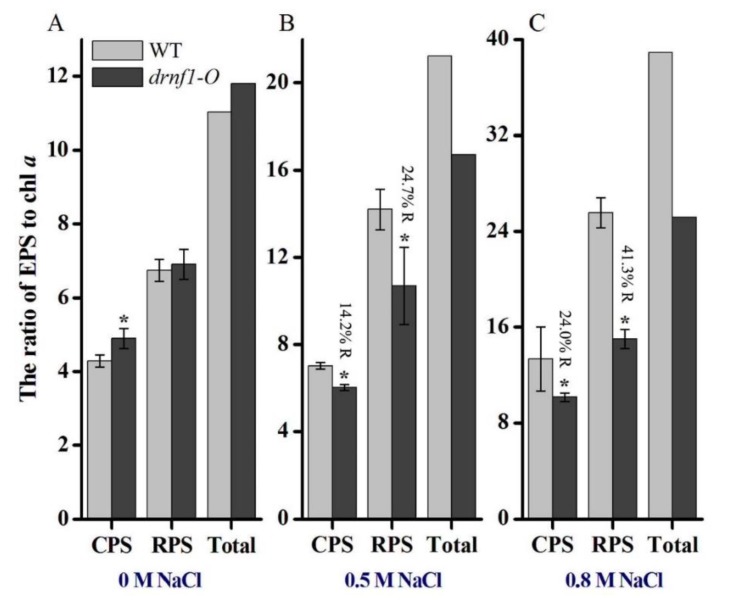
Production of total exopolysaccharide (EPS), capsular EPS (CPS), and released EPS (RPS) in WT and transgenic *Synechocystis* before (**A**) and after salt stresses (**B**,**C**). Data are mean ± s.d. (*n* = 3). Asterisks indicate significant differences (*p* < 0.05) with respect to the WT under the same conditions (Student’s *t*-test). R: the reduction (relative to the WT control).

A wide range of cyanobacteria synthesize and secrete extracellular polymeric substances, mainly of the polysaccharidic nature. EPS production is usually incurred by abiotic stress stimuli, including salt stress [35]. The changes of polysaccharide production (CPS, RPS, and total EPS) in response to salt stress were compared between WT and transgenic *Synechocystis* (Figure 4). The relative polysaccharide level was indexed by the EPS/Chl *a* ratio. In the absence of salt stress, the transgenic stain produced slightly more CPS and total EPS than the WT (Figure 4A). After subjected to 0.5 M NaCl stress, an increase in CPS (1.6 folds) and RPS (2.1 folds) as well as total EPS was observed in the WT, while the increased levels were 1.2-fold for CPS and 1.5-fold for RPS in the transgenic strain (Figure 4B). After subjected to 0.8 M NaCl stress, the increased levels of CPS and RPS were 3.1- and 3.8-fold, respectively, in the WT, while these levels were 2.1- and 2.2-fold, respectively, in the transgenic strain (Figure 4C). Thus, the increased EPS levels were lower in the transgenic strain as compared to the WT strain upon salt stress and this effect was more severe at the higher salt concentration. These results indicated that the salt-enhanced production of polysaccharides was attenuated in transgenic *Synechocystis*.

### 3.5. Transcription, K^+^ and Na^+^ Contents in Transgenic Synechocystis

**Figure 5:**
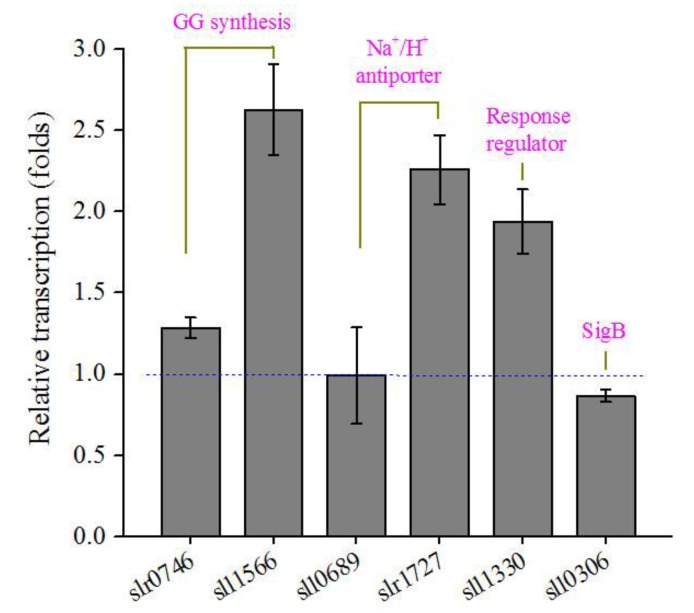
Relative transcriptional levels of six representative salt-responsive genes in transgenic *Synechocystis* upon salt stress as compared to the WT. Data are mean ± s.d. (*n* = 3).

The genes involved in the synthesis of compatible solutes, Na^+^/K^+^ ion transporter, and transcriptional regulation are crucial for the salt tolerance of cyanobacterial cells [10,46,47]. Here we investigated the transcriptional changes of several representative genes in transgenic *Synechocystis* upon salt stress (Figure 5). Their transcription levels in the transgenic strain were compared to those in the WT. GG is the major compatible solute in *Synechocystis*. Two genes (*slr0746* and *sll1566*) responsible for GG synthesis were more up-regulated in the transgenic strain. One (*slr1727*) of the two Na^+^/H^+^ antiporter genes was also more up-regulated. Sll1330 is a transcriptional regulator that controls the expression of sugar catabolic genes under light and glucose-supplemented conditions or during nitrogen starvation [48,49]. The transcription of *sll1330* was more up-regulated. The SigB (Sll0306) is involved in many salt acclimation processes, including positively regulating compatible solutes, heat shock proteins, and carotenoids [40]. However, *sll0306* was only slightly down-regulated in the transgenic strain. These results implied that transcriptional patterns of salt-responsive genes were altered in transgenic *Synechocystis*.

**Figure 6:**
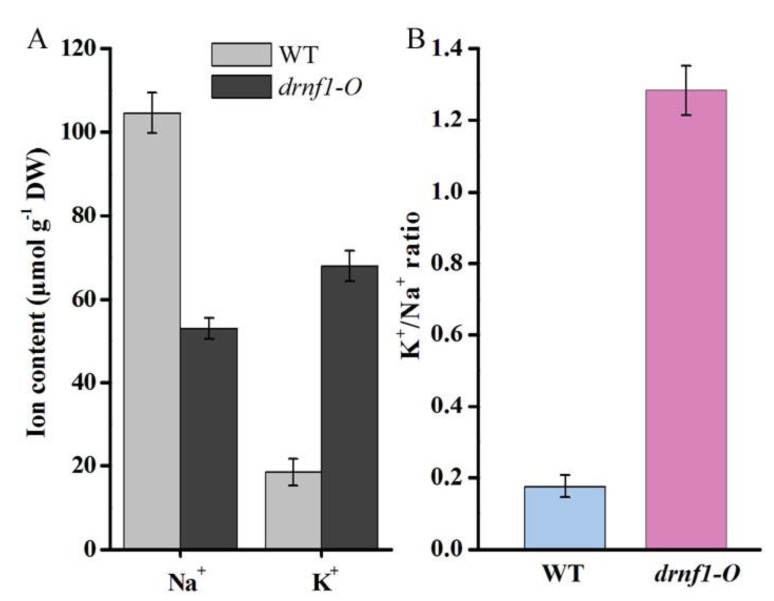
(**A**) The Na^+^ and K^+^ levels; and (**B**) K^+^/Na^+^ ratios of salt-stressed WT and transgenic *Synechocystis* strains. 0.8 M NaCl was used for the stress treatment. Data are mean ± s.d. (*n* = 3).

The basic mechanism of salt acclimation includes the active extrusion of toxic Na^+^ and import of harmless K^+^ [10]. In the present study, Na^+^ and K^+^ contents between 0.8 M NaCl-stressed WT and transgenic stains were measured (Figure 6). The Na^+^ level was obviously lowered (approximately 50% reduction) in transgenic strain, while the K^+^ level was obviously increased (approximately 3.7-fold) (Figure 6A). A much higher K^+^/Na^+^ ratio (1.28) occurred in the transgenic strain, being more than 7-fold that (0.17) of the WT (Figure 6B). It was previously reported that at extracellular NaCl concentrations of 2, 342, and 684 mM, the intracellular K^+^/Na^+^ ratios were 1.84, 0.81, and 0.69, respectively, in *Synechocystis* [50]. A high cytosolic K^+^/Na^+^ ratio is crucial for cell survival under salt stress [10,47,51]. Thus, a significant further improvement of K^+^/Na^+^ homeostasis, one of the most important aspects for salt acclimation, was achieved in transgenic *Synechocystis* upon salt stress.

### 3.6. Increased Salt Tolerance in Transgenic drnf1 Arabidopsis

**Figure 7:**
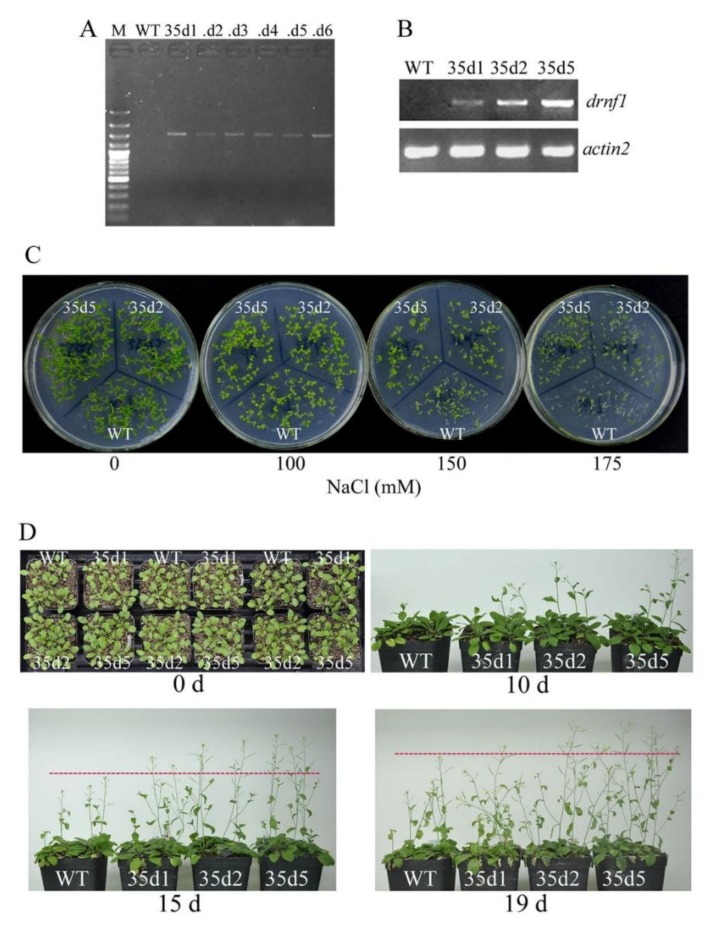
Identification of transgenic *Arabidopsis* plants and their salt tolerance phenotypes. (**A**) Identification of transgenes by PCR amplification of genomic DNA using *drnf1* gene-specific primers; (**B**) Semi-quantitative RT-PCR analysis of *drnf1* transcription. M: B031 DNA ladder (Dingguo, Beijing, China). WT: wild-type plant. 35d1-6: independent transgenic lines; (**C**) Germination performance of transgenic seeds on solid media supplemented with various concentrations of NaCl; (**D**) Growth performance of transgenic plants in 150 mM NaCl solution-watered soils. Three-week-old plantlets (0 d) were subjected to salt stress treatment.

To evaluate whether the *drnf1* gene could confer salt tolerance in higher plants, we employed the model plant *Arabidopsis* to generate transgenic lines for assay. The transgene was verified by PCR amplification of genomic DNA (Figure 7A). Three transgenic lines that showed different expression levels of *drnf1* (Figure 7B) were chosen for phenotypic observation. Seed germination performance of transgenic lines upon salt stress was first compared with that of WT (Figure 7C). In the plates without salt stress and with 100 mM NaCl, seeds of two transgenic lines (35d2 and 35d5) and WT all showed 100% germination rates two weeks after germination; however, in the latter condition, approximately 10.9% of WT seedlings appeared smaller. Along with the increase of salt stress (150 and 175 mM NaCl), WT seeds showed more severe inhibition in germination than transgenic seeds. The germination rate of WT was ~32.7% at 150 mM NaCl, while those of 35d2 and 35d5 seeds were ~60.0% and 69.1%, respectively; at 175 mM NaCl, the germination rate of WT is ~7.3%, while those of 35d2 and 35d5 seeds were ~27.2% and 43.6%, respectively. Plant growth of transgenic lines (35d1, 35d2 and 35d5) upon salt stress was also compared to that of WT (Figure 7D). Three-week old plantlets with similar sizes were used to salt stress through watering the soils with 150 mM NaCl solution as described in the Methods. The shoot elongation was the most easily observed phenotype in this study. After salt stress for 10 days, rapider bolting was observed in transgenic plants. After salt stress for 15 days and 19 days, the main shoots of transgenic plants were still overall higher than those of WT plants; also, transgenic plants showed longer or flourishing lateral shoots. We detected K^+^ and Na^+^ contents in leaves of transgenic and WT plants but did find a better K^+^/Na^+^ ratio in the former; however, proline levels were much more higher in the former (data not shown). Salinity often triggers the alteration of proline content and its over-accumulation can serve as an indicator of enhanced stress tolerance [52]. Generally, these results suggested that the *drnf1* gene possessed the potential of conferring salt tolerance in higher plants.

## 4. Discussion

Because of long-term adaptation in harsh environments, *N. flagelliforme* should have evolved with a precious gene resource with regard to abiotic stress resistance. The significant accumulation of both trehalose and sucrose in response to various stresses was found in *N. flagelliforme* [13], which is correlated with salt tolerance [46]. Although this species is mainly drought-adapted, it exhibited a well adaptive flexibility to salt addition and removal. This feature is compatible to the extremely changeable moisture environments in native habitats. The removal of salt stress (0.2 or 0.4 M NaCl) also seems to incur better recovery, implying a possible activation of salt adaptation systems in this species. Except for the formerly reported genes [16], a newly identified salt-tolerant gene *drnf1* from *N. flagelliforme* also proves that this species serves as an important gene resource. Considering that the *drnf1* gene could confer salt tolerance in *E. coli* (in our preliminary screening), *Synechocystis* and *Arabidopsis*, it should present a novel gene with relatively universal application potential.

In the present study, the potential mechanism of *drnf1* in conferring salt tolerance was mainly discussed in transgenic *Synechocystis*. The basal salt acclimation strategy includes two principal reactions, the active export of toxic ions and the accumulation of compatible solutes [47]. We chose several important genes to evaluate their salt stress-incurred transcriptional alterations in transgenic strain, relative to WT strain. Two genes related with GG-synthesis and one Na^+^/H^+^ transporter gene were up-regulated at a higher level in the transgenic strain. The K^+^/Na^+^ homeostasis was also found to maintain a higher level upon salt stress in the transgenic strain, suggestive of a good regulation of this basal mechanism. However, we did not detect a higher accumulation of intracellular GG content in salt-stressed transgenic strain. Intracellular GG level is also regulated by SigB [40] and other genes including its re-assimilation gene *slr1670* [53]. Therefore, the final accumulation of the compatible solute GG may not exert a dominant role in improving salt tolerance in the transgenic strain. The higher up-regulation of the sugar catabolism-related *sll1330* [48,49] was observed in the transgenic strain, which implies a potentially positive production of energy and metabolites for stress resistance. These results gave the implication that DRNF1 could affect the basal salt acclimation process in transgenic hosts, resulting in a combined result of increased salt tolerance. 

Cyanobacterial EPS was reported to play a role in abiotic stress tolerance, through eliminating the induced reactive oxygen species [54,55]. However, it mainly exerts the extracellular anti-oxidative role and this capability in salt tolerance is limited. In this study, the EPS was less produced in the transgenic strain upon salt stress as compared to the WT. This implies that more energy would be saved because of the lower accumulation of EPS. Upon salt stress, cyanobacteria require considerable energy expenditure to get rid of Na^+^ from the cytoplasm [10]. Thus, the saved energy would be utilized to facilitate Na^+^ transport. In addition, the active cellular metabolism upon salt stress was reflected by the enhanced respiration and also suggested by the above-mentioned up-regulation of the *sll1330* gene. The elevated respiration rate was responsible for maintaining the transmembrane H^+^ gradient that energizes Na^+^/H^+^ antiporters [56]. Therefore, DRNF1 might also be involved in facilitating the energy utilization for salt tolerance in transgenic hosts.

The most important aspect for functional genes is their potential application in crops. We preliminarily evaluated this application possibility by overexpressing the *drnf1* gene in the *Arabidopsis* plant. The phenotypic results indicated that this gene could confer salt tolerance in seed germination and plant growth levels. Unlike in transgenic *Synechocystis*, we did not detect an elevated K^+^/Na^+^ ratio in the leaves of transgenic plants. Under saline conditions plants can either restrict the excess Na^+^ in the vacuole or compartmentalize it in different tissues to minimize the damage [57]. Thus, it is not excluded that excess Na^+^ was compartmentalized into the vacuole in transgenic *Arabidopsis*. From an application point of view, those positive phenotypes are already suggestive of a good prospect of this gene in transgenic plants.

In summary, a new prokaryotic gene *drnf1* from *N. flagelliforme* was identified and characterized as having a salt tolerant function, which will enrich the candidate gene pool for plant genetic engineering. Its biochemical feature and concrete molecular function remain to be explored in future studies.

## Figures and Tables

**Table 1:** Net photosynthesis and respiration of WT and *drnf1*-O *Synechocystis* strains before and after salt stress measured by O_2_ evolution.

Sample	Net Photosynthesis (µmol h^−1^ mg^−1^ Chl *a*)	Respiration (µmol h^−1^ mg^−1^ Chl *a*)
WT	90.5 ± 11.1	38.6 ± 9.1
*drnf1-O*	105.6 ± 13.7	68.0 ± 12.5 *
WT +	60.3 ± 10.3	28.1 ± 5.3
*drnf1-O* +	70.1 ± 10.5	53.0 ± 4.1 *

+: indicates the treatment by 0.5 M NaCl for 2.5 h. Data shown are means ± s.d. (*n* = 3). Asterisks indicate significant differences (*p* < 0.05) with respect to the WT under the same conditions (Student’s *t*-test).
